# Supplementary material for: Nectar robbing by the invasive bumblebee Bombus terrestris (Apidae) changes the behavior of native flower visitors of Fuchsia magellanica Lam. (Onagraceae) but not seed set
Source: PeerJ. 2025 Oct 22;13:e20253. doi: 10.7717/peerj.20253 (PMC12553366; doi:10.7717/peerj.20253)
Supplement: Supplemental Information 2 — Mean percentage (±1 SE) of Fuchsia magellanica flowers pierced by Bombus terrestris (primary nectar robbing) in 24 populations across the Los Ríos and Los Lagos regions of southern Chile. Populations are listed in alphabetical order and correspond to the numbering used in Figure 1 [file peerj-13-20253-s002.docx]

**Appendix 1.** Mean percentage (± 1 SE) of *Fuchsia magellanica* flowers pierced by *Bombus terrestris* (primary nectar robbing) across the 24 study populations in the Los Ríos and Los Lagos regions of Chile. Populations are listed in alphabetical order and correspond to the numbering used in Figure 1.

| N° | Population | Latitude | Longitude | Altitude (m a.s.l.) | Region | Primary nectar robbing (%) (Mean ± 1SE) |
| --- | --- | --- | --- | --- | --- | --- |
| 1 | Alerce Andino | 41°17′18″ S | 73°24′42″ W | 1106 | Los Lagos | 0.5 ± 0.5 |
| 2 | Cardenal Samoré | 40°39′40″ S | 72°07′48″ W | 536 | Los Lagos | 0.5 ± 0.5 |
| 3 | Chacao | 41°49′03″ S | 73°31′39″ W | 24 | Los Lagos | 54.5 ± 6.0 |
| 4 | Chaitén | 42°57′16″ S | 72°38′28″ W | 320 | Los Lagos | 2.0 ± 1.2 |
| 5 | Contao | 41°57′41″ S | 72°40′46″ W | 186 | Los Lagos | 0.0 ± 0.0 |
| 6 | Cutipay | 39°51′30″ S | 73°19′59″ W | 51 | Los Ríos | 94.0 ± 2.5 |
| 7 | Futaleufú | 43°11′17″ S | 71°51′55″ W | 647 | Los Lagos | 53.0 ± 9.3 |
| 8 | Hornopirén | 41°56′54″ S | 72°27′09″ W | 369 | Los Lagos | 0.0 ± 0.0 |
| 9 | Llanquihue | 41°14′17″ S | 73°01′39″ W | 119 | Los Lagos | 97.0 ± 1.5 |
| 10 | Los Hualles | 40°26′39″ S | 73°23′43″ W | 236 | Los Lagos | 11.5 ± 6.3 |
| 11 | Los Muermos | 41°23′59″ S | 73°28′43″ W | 172 | Los Lagos | 0.0 ± 0.0 |
| 12 | Los Venados | 40°20′47″ S | 73°04′47″ W | 339 | Los Ríos | 0.0 ± 0.0 |
| 13 | Nercón | 42°12′38″ S | 73°47′53″ W | 57 | Los Lagos | 14.5 ± 3.8 |
| 14 | Puente Dumontt | 43°18′42″ S | 71°59′21″ W | 426 | Los Lagos | 0.0 ± 0.0 |
| 15 | Puerto Cárdenas | 42°47′37″ S | 72°52′17″ W | 174 | Los Lagos | 0.0 ± 0.0 |
| 16 | Pto. Fuy | 40°09′54″ S | 71°54′09″ W | 719 | Los Ríos | 6.0 ± 3.4 |
| 17 | Puerto Octay | 40°56′43″ S | 72°51′03″ W | 111 | Los Lagos | 0.0 ± 0.0 |
| 18 | Puntra | 42°05′49″ S | 73°43′22″ W | 12 | Los Lagos | 4.0 ± 1.4 |
| 19 | Puyehue | 40°40′25″ S | 72°58′02″ W | 565 | Los Lagos | 0.5 ± 0.5 |
| 20 | Quellón 1 | 43°07′33″ S | 73°39′13″ W | 98 | Los Lagos | 20.0 ± 5.0 |
| 21 | Quellón 2 | 43°07′35″ S | 73°39′38″ W | 156 | Los Lagos | 6.0 ± 3.2 |
| 22 | Santa Bárbara | 40°09′59″ S | 72°47′21″ W | 181 | Los Lagos | 4.5 ± 2.2 |
| 23 | Valdivia | 39°48′05″ S | 73°14′48″ W | 99 | Los Ríos | 93.5 ± 4.6 |
| 24 | Yerba Loza | 42°50′27″ S | 73°45′44″ W | 77 | Los Lagos | 0.0 ± 0.0 |
