# Supplementary material for: Nectar robbing by the invasive bumblebee Bombus terrestris (Apidae) changes the behavior of native flower visitors of Fuchsia magellanica Lam. (Onagraceae) but not seed set
Source: PeerJ. 2025 Oct 22;13:e20253. doi: 10.7717/peerj.20253 (PMC12553366; doi:10.7717/peerj.20253)
Supplement: Supplemental Information 3 — Mean nectar production rate (μl/flower/day) and standing crop of nectar (μl/flower) in pierced and undamaged flowers of Fuchsia magellanica across 24 populations in southern Chile. Values are means ± 1 SE. “0” indicates flowers measured but with no nectar present; “nr” indicates that no pierced flowers were present in the population, so no measurements could be taken [file peerj-13-20253-s003.docx]

**Appendix 2.** Nectar production rate (µl/flower/day) and standing crop of nectar (µl/flower) in pierced and undamaged flowers of *Fuchsia magellanica* across 24 populations in southern Chile. Values are means ± 1 SE. “0” indicates flowers measured with no nectar present; “nr” indicates that no pierced flowers were present in the population, so no measurements could be taken.

| Population | Nectar production rate (µl of nectar / flower / day) | | | | | | Standing crop of néctar (µl of nectar / flower) | | | | | |
| --- | --- | --- | --- | --- | --- | --- | --- | --- | --- | --- | --- | --- |
|  | Healthy flowers | | | Pierced flowers | | | Healthy flowers | | | Pierced flowers | | |
| Alerce Andino | 18.4 | ± | 1.8 |  | nr |  | 0.7 | ± | 0.2 |  | nr |  |
| Cardenal Samoré | 46.0 | ± | 2.8 |  | nr |  | 38.4 | ± | 2.1 |  | nr |  |
| Chacao | 28.0 | ± | 2.3 | 5.1 | ± | 1.1 | 6.1 | ± | 1.1 | 1.6 | ± | 0.7 |
| Chaitén | 22.6 | ± | 8.7 | 0.0 | ± | 4.4 | 16.9 | ± | 4.3 | 8.9 | ± | 2.7 |
| Contao | 17.8 | ± | 1.7 |  | nr |  | 1.7 | ± | 0.2 |  | nr |  |
| Cutipay | 3.0 | ± | 0.7 |  | nr |  | 0.1 | ± | 0.0 |  | nr |  |
| Futaleufú | 8.0 | ± | 6.2 | 3.4 | ± | 3.1 | 1.0 | ± | 3.0 | 0.0 | ± | 1.9 |
| Hornopirén | 7.9 | ± | 1.1 |  | nr |  | 4.0 | ± | 0.5 |  | nr |  |
| Llanquihue | 3.7 | ± | 8.7 | 5.7 | ± | 4.4 | 0.0 | ± | 4.3 | 0.0 | ± | 2.7 |
| Los Hualles | 6.6 | ± | 5.0 | 1.2 | ± | 2.5 | 1.7 | ± | 2.5 | 0.3 | ± | 1.6 |
| Los Muermos | 6.6 | ± | 1.3 |  | nr |  | 1.2 | ± | 0.3 |  | nr |  |
| Los Venados | 23.4 | ± | 2.4 |  | nr |  | 9.7 | ± | 1.0 |  | nr |  |
| Nercón | 8.8 | ± | 5.0 | 6.6 | ± | 2.5 | 10.3 | ± | 2.5 | 8.1 | ± | 1.6 |
| Puente Dumontt | 7.0 | ± | 0.9 |  | nr |  | 0.8 | ± | 0.2 |  | nr |  |
| Puerto Cárdenas | 9.2 | ± | 1.3 |  | nr |  | 1.4 | ± | 0.4 |  | nr |  |
| Puerto Fuy | 15.8 | ± | 5.0 | 5.7 | ± | 2.5 | 4.9 | ± | 2.5 | 0.0 | ± | 1.6 |
| Puerto Octay | 13.3 | ± | 1.1 |  | nr |  | 6.6 | ± | 0.8 |  | nr |  |
| Puntra | 3.7 | ± | 6.2 | 2.7 | ± | 3.1 | 3.1 | ± | 3.0 | 0.0 | ± | 1.9 |
| Puyehue | 20.2 | ± | 2.2 |  | nr |  | 10.9 | ± | 1.5 |  | nr |  |
| Quellón 1 | 10.6 | ± | 3.1 | 1.3 | ± | 1.5 | 4.6 | ± | 1.5 | 1.3 | ± | 1.0 |
| Quellón 2 | 5.3 | ± | 1.5 |  | nr |  | 10.5 | ± | 1.2 |  | nr |  |
| Santa Bárbara | 21.1 | ± | 1.9 |  | nr |  | 16.0 | ± | 1.8 |  | nr |  |
| Valdivia | 3.1 | ± | 0.7 |  | nr |  | 0.2 | ± | 0.1 |  | nr |  |
| Yerba Loza | 5.0 | ± | 0.7 |  | nr |  | 2.9 | ± | 0.4 |  | nr |  |
