# Supplementary material for: Nectar robbing by the invasive bumblebee Bombus terrestris (Apidae) changes the behavior of native flower visitors of Fuchsia magellanica Lam. (Onagraceae) but not seed set
Source: PeerJ. 2025 Oct 22;13:e20253. doi: 10.7717/peerj.20253 (PMC12553366; doi:10.7717/peerj.20253)
Supplement: Supplemental Information 4 — Mean (±1 SE) rates of pollinating and robbing visits (number of visits per flower per 10 min) and mean visitation frequencies (%) by the hummingbird Sephanoides sephaniodes , the native bumblebee Bombus dahlbomii , the exotic bumblebee B. terrestris , and the exotic honeybee Apis mellifera across 24 populations of Fuchsia magellanica in southern Chile [file peerj-13-20253-s004.docx]

**Appendix 3**. Foraging behavior of floral visitors to *Fuchsia magellanica*. Mean (± SE) rates of pollinating and robbing visits (number of visits per flower per 10 min) and mean visitation frequencies (%) by the hummingbird *Sephanoides sephaniodes*, the native bumblebee *Bombus dahlbomii*, the exotic bumblebee *B. terrestris*, and the exotic honeybee *Apis mellifera* across 24 study sites in southern Chile.

| Foraging behavior | Rate of visits (N° of visits / flower / 10 min) | | | | | | | | | | | | | | |
| --- | --- | --- | --- | --- | --- | --- | --- | --- | --- | --- | --- | --- | --- | --- | --- |
|  | *S. sephaniodes* | | | *B. dahlbomii* | | | *B. terrestris* | | | *A. mellifera* | | | All animals | | |
| Pollinating visits: |  |  |  |  |  |  |  |  |  |  |  |  |  |  |  |
| Alerce Andino | 0.069 | ± | 0.033 | 0.003 | ± | 0.003 | 0.000 | ± | 0.000 | 0.172 | ± | 0.052 | 0.243 | ± | 0.059 |
| Cardenal Samoré | 0.003 | ± | 0.003 | 0.000 | ± | 0.000 | 0.000 | ± | 0.000 | 0.000 | ± | 0.000 | 0.003 | ± | 0.003 |
| Chacao | 0.000 | ± | 0.000 | 0.014 | ± | 0.014 | 0.000 | ± | 0.000 | 0.008 | ± | 0.008 | 0.023 | ± | 0.017 |
| Chaitén | 0.000 | ± | 0.000 | 0.005 | ± | 0.004 | 0.043 | ± | 0.025 | 0.000 | ± | 0.000 | 0.048 | ± | 0.026 |
| Contao | 0.016 | ± | 0.011 | 0.026 | ± | 0.019 | 0.000 | ± | 0.000 | 0.000 | ± | 0.000 | 0.042 | ± | 0.021 |
| Cutipay | 0.000 | ± | 0.000 | 0.000 | ± | 0.000 | 0.000 | ± | 0.000 | 0.003 | ± | 0.003 | 0.003 | ± | 0.003 |
| Futaleufú | 0.037 | ± | 0.025 | 0.004 | ± | 0.004 | 0.000 | ± | 0.000 | 0.000 | ± | 0.000 | 0.040 | ± | 0.025 |
| Hornopirén | 0.000 | ± | 0.000 | 0.000 | ± | 0.000 | 0.000 | ± | 0.000 | 0.004 | ± | 0.004 | 0.004 | ± | 0.004 |
| Llanquihue | 0.000 | ± | 0.000 | 0.001 | ± | 0.001 | 0.004 | ± | 0.004 | 0.000 | ± | 0.000 | 0.005 | ± | 0.004 |
| Los Hualles | 0.000 | ± | 0.000 | 0.008 | ± | 0.006 | 0.013 | ± | 0.013 | 0.000 | ± | 0.000 | 0.021 | ± | 0.014 |
| Los Muermos | 0.000 | ± | 0.000 | 0.051 | ± | 0.025 | 0.000 | ± | 0.000 | 0.000 | ± | 0.000 | 0.051 | ± | 0.025 |
| Los Venados | 0.002 | ± | 0.002 | 0.000 | ± | 0.000 | 0.010 | ± | 0.010 | 0.008 | ± | 0.004 | 0.020 | ± | 0.011 |
| Nercón | 0.020 | ± | 0.014 | 0.000 | ± | 0.000 | 0.000 | ± | 0.000 | 0.000 | ± | 0.000 | 0.020 | ± | 0.015 |
| Puente Dumontt | 0.027 | ± | 0.023 | 0.072 | ± | 0.033 | 0.000 | ± | 0.000 | 0.000 | ± | 0.000 | 0.100 | ± | 0.041 |
| Puerto Cárdenas | 0.003 | ± | 0.003 | 0.043 | ± | 0.015 | 0.020 | ± | 0.014 | 0.000 | ± | 0.000 | 0.066 | ± | 0.021 |
| Puerto Fuy | 0.023 | ± | 0.014 | 0.036 | ± | 0.018 | 0.007 | ± | 0.007 | 0.004 | ± | 0.004 | 0.071 | ± | 0.023 |
| Puerto Octay | 0.000 | ± | 0.000 | 0.017 | ± | 0.012 | 0.000 | ± | 0.000 | 0.048 | ± | 0.027 | 0.064 | ± | 0.030 |
| Puntra | 0.000 | ± | 0.000 | 0.024 | ± | 0.020 | 0.000 | ± | 0.000 | 0.000 | ± | 0.000 | 0.024 | ± | 0.021 |
| Puyehue | 0.029 | ± | 0.022 | 0.000 | ± | 0.000 | 0.000 | ± | 0.000 | 0.030 | ± | 0.014 | 0.059 | ± | 0.025 |
| Quellón 1 | 0.000 | ± | 0.000 | 0.005 | ± | 0.005 | 0.000 | ± | 0.000 | 0.000 | ± | 0.000 | 0.005 | ± | 0.005 |
| Quellón 2 | 0.000 | ± | 0.000 | 0.000 | ± | 0.000 | 0.000 | ± | 0.000 | 0.000 | ± | 0.000 | 0.000 | ± | 0.000 |
| Santa Bárbara | 0.000 | ± | 0.000 | 0.000 | ± | 0.000 | 0.000 | ± | 0.000 | 0.000 | ± | 0.000 | 0.000 | ± | 0.000 |
| Valdivia | 0.000 | ± | 0.000 | 0.000 | ± | 0.000 | 0.000 | ± | 0.000 | 0.000 | ± | 0.000 | 0.000 | ± | 0.000 |
| Yerba Loza | 0.004 | ± | 0.004 | 0.000 | ± | 0.000 | 0.000 | ± | 0.000 | 0.000 | ± | 0.000 | 0.004 | ± | 0.004 |
| Mean rate | 0.010 | ± | 0.012 | 0.013 | ± | 0.012 | 0.004 | ± | 0.007 | 0.012 | ± | 0.013 | 0.038 | ± | 0.005 |
| Mean frequency (%) | 11.9 | ± | 18.9 | 15.8 | ± | 19.4 | 5.0 | ± | 11.1 | 14.2 | ± | 20.2 | 46.9 | ± | 6.3 |
| Robbing visits: |  |  |  |  |  |  |  |  |  |  |  |  |  |  |  |
| Alerce Andino | 0.000 | ± | 0.000 | 0.000 | ± | 0.000 | 0.000 | ± | 0.000 | 0.000 | ± | 0.000 | 0.000 | ± | 0.000 |
| Cardenal Samoré | 0.000 | ± | 0.000 | 0.000 | ± | 0.000 | 0.000 | ± | 0.000 | 0.000 | ± | 0.000 | 0.000 | ± | 0.000 |
| Chacao | 0.000 | ± | 0.000 | 0.000 | ± | 0.000 | 0.032 | ± | 0.014 | 0.008 | ± | 0.008 | 0.039 | ± | 0.017 |
| Chaitén | 0.000 | ± | 0.000 | 0.000 | ± | 0.000 | 0.000 | ± | 0.000 | 0.000 | ± | 0.000 | 0.000 | ± | 0.000 |
| Contao | 0.000 | ± | 0.000 | 0.000 | ± | 0.000 | 0.000 | ± | 0.000 | 0.000 | ± | 0.000 | 0.000 | ± | 0.000 |
| Cutipay | 0.000 | ± | 0.000 | 0.000 | ± | 0.000 | 0.328 | ± | 0.077 | 0.008 | ± | 0.008 | 0.336 | ± | 0.084 |
| Futaleufú | 0.000 | ± | 0.000 | 0.000 | ± | 0.000 | 0.042 | ± | 0.015 | 0.000 | ± | 0.000 | 0.042 | ± | 0.015 |
| Hornopirén | 0.000 | ± | 0.000 | 0.000 | ± | 0.000 | 0.000 | ± | 0.000 | 0.000 | ± | 0.000 | 0.000 | ± | 0.000 |
| Llanquihue | 0.000 | ± | 0.000 | 0.015 | ± | 0.011 | 0.195 | ± | 0.049 | 0.000 | ± | 0.000 | 0.210 | ± | 0.050 |
| Los Hualles | 0.000 | ± | 0.000 | 0.023 | ± | 0.016 | 0.009 | ± | 0.006 | 0.000 | ± | 0.000 | 0.032 | ± | 0.023 |
| Los Muermos | 0.000 | ± | 0.000 | 0.000 | ± | 0.000 | 0.000 | ± | 0.000 | 0.000 | ± | 0.000 | 0.000 | ± | 0.000 |
| Los Venados | 0.000 | ± | 0.000 | 0.000 | ± | 0.000 | 0.000 | ± | 0.000 | 0.000 | ± | 0.000 | 0.000 | ± | 0.000 |
| Nercón | 0.000 | ± | 0.000 | 0.000 | ± | 0.000 | 0.000 | ± | 0.000 | 0.000 | ± | 0.000 | 0.000 | ± | 0.000 |
| Puente Dumontt | 0.000 | ± | 0.000 | 0.000 | ± | 0.000 | 0.000 | ± | 0.000 | 0.000 | ± | 0.000 | 0.000 | ± | 0.000 |
| Puerto Cárdenas | 0.000 | ± | 0.000 | 0.000 | ± | 0.000 | 0.000 | ± | 0.000 | 0.000 | ± | 0.000 | 0.000 | ± | 0.000 |
| Puerto Fuy | 0.000 | ± | 0.000 | 0.000 | ± | 0.000 | 0.027 | ± | 0.013 | 0.000 | ± | 0.000 | 0.027 | ± | 0.014 |
| Puerto Octay | 0.000 | ± | 0.000 | 0.000 | ± | 0.000 | 0.000 | ± | 0.000 | 0.000 | ± | 0.000 | 0.000 | ± | 0.000 |
| Puntra | 0.000 | ± | 0.000 | 0.000 | ± | 0.000 | 0.000 | ± | 0.000 | 0.000 | ± | 0.000 | 0.000 | ± | 0.000 |
| Puyehue | 0.000 | ± | 0.000 | 0.000 | ± | 0.000 | 0.000 | ± | 0.000 | 0.000 | ± | 0.000 | 0.000 | ± | 0.000 |
| Quellón 1 | 0.000 | ± | 0.000 | 0.000 | ± | 0.000 | 0.000 | ± | 0.000 | 0.000 | ± | 0.000 | 0.000 | ± | 0.000 |
| Quellón 2 | 0.000 | ± | 0.000 | 0.000 | ± | 0.000 | 0.000 | ± | 0.000 | 0.000 | ± | 0.000 | 0.000 | ± | 0.000 |
| Santa Bárbara | 0.000 | ± | 0.000 | 0.000 | ± | 0.000 | 0.000 | ± | 0.000 | 0.000 | ± | 0.000 | 0.000 | ± | 0.000 |
| Valdivia | 0.000 | ± | 0.000 | 0.000 | ± | 0.000 | 0.342 | ± | 0.089 | 0.006 | ± | 0.006 | 0.348 | ± | 0.090 |
| Yerba Loza | 0.000 | ± | 0.000 | 0.000 | ± | 0.000 | 0.000 | ± | 0.000 | 0.000 | ± | 0.000 | 0.000 | ± | 0.000 |
| Mean rate | 0.000 | ± | 0.000 | 0.002 | ± | 0.004 | 0.041 | ± | 0.027 | 0.001 | ± | 0.003 | 0.043 | ± | 0.007 |
| Mean frequency (%) | 0.0 | ± | 0.0 | 2.0 | ± | 0.0 | 49.9 | ± | 0.1 | 1.1 | ± | 0.0 | 53.1 | ± | 8.6 |
