# Supplementary material for: Nectar robbing by the invasive bumblebee Bombus terrestris (Apidae) changes the behavior of native flower visitors of Fuchsia magellanica Lam. (Onagraceae) but not seed set
Source: PeerJ. 2025 Oct 22;13:e20253. doi: 10.7717/peerj.20253 (PMC12553366; doi:10.7717/peerj.20253)
Supplement: Supplemental Information 5 — Mean seed set (number of seeds per flower ± 1 SE) of Fuchsia magellanica across 24 populations in southern Chile. Experimental conditions included: absence of pollinators and robbers (−Pol −Rob), presence of pollinators without robbers (+Pol −Rob), and presence of both pollinators and robbers (+Pol +Rob) [file peerj-13-20253-s005.docx]

**Appendix 4.** Seed set of *Fuchsia magellanica* across 24 populations in southern Chile under different experimental conditions: absence of pollinators and robbers (−Pol −Rob), presence of pollinators without robbers (+Pol −Rob), and presence of both pollinators and robbers (+Pol +Rob). Values represent means ± 1 SE (number of seeds per flower).

| Population | Seed set (N° of seeds / flower) | | | | | | | | |
| --- | --- | --- | --- | --- | --- | --- | --- | --- | --- |
|  | -Rob -Pol | | | -Rob +Pol | | | +Rob +Pol | | |
| Alerce Andino | 6.2 | ± | 4.0 | 22.0 | ± | 8.2 | 97.9 | ± | 23.9 |
| Cardenal Samoré | 4.2 | ± | 2.2 | 13.0 | ± | 8.1 | 43.5 | ± | 12.3 |
| Chacao | 8.2 | ± | 3.6 | 24.0 | ± | 12.5 | 9.5 | ± | 6.6 |
| Chaitén | 5.3 | ± | 4.9 | 2.0 | ± | 1.8 | 7.6 | ± | 4.8 |
| Contao | 0.0 | ± | 0.0 | 37.1 | ± | 11.5 | 85.8 | ± | 18.7 |
| Cutipay | 14.8 | ± | 5.6 | 24.5 | ± | 9.6 | 43.1 | ± | 14.9 |
| Futaleufú | 1.5 | ± | 1.0 | 0.0 | ± | 0.0 | 24.9 | ± | 9.4 |
| Hornopirén | 2.6 | ± | 2.6 | 21.1 | ± | 7.8 | 50.7 | ± | 19.3 |
| Llanquihue | 16.6 | ± | 7.5 | 42.9 | ± | 11.1 | 35.9 | ± | 10.6 |
| Los Hualles | 25.7 | ± | 14.0 | 163.2 | ± | 17.8 | 119.5 | ± | 20.0 |
| Los Muermos | 31.7 | ± | 15.6 | 82.6 | ± | 18.5 | 52.8 | ± | 12.3 |
| Los Venados | 0.3 | ± | 0.3 | 61.1 | ± | 15.6 | 83.7 | ± | 18.1 |
| Nercón | 15.6 | ± | 14.0 | 31.0 | ± | 9.9 | 25.7 | ± | 9.3 |
| Puente Dumontt | 0.0 | ± | 0.0 | 75.6 | ± | 17.4 | 9.3 | ± | 8.2 |
| Puerto Cárdenas | 0.0 | ± | 0.0 | 35.9 | ± | 14.2 | 13.7 | ± | 5.2 |
| Puerto Fuy | 8.9 | ± | 5.5 | 25.1 | ± | 9.3 | 86.1 | ± | 25.0 |
| Puerto Octay | 35.2 | ± | 17.0 | 106.6 | ± | 17.2 | 69.2 | ± | 21.3 |
| Puntra | 1.1 | ± | 0.8 | 30.2 | ± | 15.8 | 22.1 | ± | 7.5 |
| Puyehue | 8.5 | ± | 5.3 | 19.7 | ± | 10.3 | 52.4 | ± | 13.6 |
| Quellón 1 | 0.0 | ± | 0.0 | 33.9 | ± | 8.6 | 21.7 | ± | 13.0 |
| Quellón 2 | 0.5 | ± | 0.5 | 8.3 | ± | 3.7 | 28.8 | ± | 12.1 |
| Santa Bárbara | 1.0 | ± | 1.0 | 11.3 | ± | 3.7 | 36.2 | ± | 10.6 |
| Valdivia | 10.0 | ± | 4.2 | 8.8 | ± | 4.2 | 12.3 | ± | 8.4 |
| Yerba Loza | 2.1 | ± | 2.1 | 21.0 | ± | 7.2 | 25.9 | ± | 7.7 |
